# Engineering Copper Iodide (CuI) for Multifunctional p‐Type Transparent Semiconductors and Conductors

**DOI:** 10.1002/advs.202100546

**Published:** 2021-05-11

**Authors:** Ao Liu, Huihui Zhu, Myung‐Gil Kim, Junghwan Kim, Yong‐Young Noh

**Affiliations:** ^1^ Department of Chemical Engineering Pohang University of Science and Technology (POSTECH) Pohang Gyeongbuk 37673 Republic of Korea; ^2^ School of Advanced Materials Science and Engineering Sungkyunkwan University Suwon 16419 Republic of Korea; ^3^ Materials Research Center for Element Strategy Tokyo Institute of Technology Mailbox SE‐6, 4259 Nagatsuta, Midori‐ku Yokohama 226‐8503 Japan

**Keywords:** inorganic p‐type semiconductors, thin‐film transistors, copper iodide, transparent conductors

## Abstract

Developing transparent p‐type semiconductors and conductors has attracted significant interest in both academia and industry because metal oxides only show efficient n‐type characteristics at room temperature. Among the different candidates, copper iodide (CuI) is one of the most promising p‐type materials because of its widely adjustable conductivity from transparent electrodes to semiconducting layers in transistors. CuI can form thin films with high transparency in the visible light region using various low‐temperature deposition techniques. This progress report aims to provide a basic understanding of CuI‐based materials and recent progress in the development of various devices. The first section provides a brief introduction to CuI with respect to electronic structure, defect states, charge transport physics, and overviews the CuI film deposition methods. The material design concepts through doping/alloying approaches to adjust the optoelectrical properties are also discussed in the first section. The following section presents recent advances in state‐of‐the‐art CuI‐based devices, including transparent electrodes, thermoelectric devices, p–n diodes, p‐channel transistors, light emitting diodes, and solar cells. In conclusion, current challenges and perspective opportunities are highlighted.

## Graphical abstract

Copper iodide (CuI) is one of the most promising p‐type materials due to its widely adjustable conductivity from conductor to semiconductor, full transparency, and easy synthesis using diverse deposition techniques. Given the rapid progress, this article provides a comprehensive understanding on CuI and the latest advances in related devices. The challenges and prospective on further development are also highlighted.

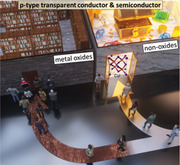

## 1 Introduction

In the past decades, inorganic transparent semiconductors and conductors (TS/C) have made great progress in modern optoelectronic research and have been widely used from charge transport layers in semiconducting devices to transparent electrodes for various optoelectronic devices.^[^ ^1^, ^2^, ^3^, ^4^, ^5^ ^]^ Currently, various products are commercialized using metal oxide TS/C. For example, Sn‐doped In_2_O_3_ (ITO), Ga or Al‐doped ZnO, and fluorine tin oxide are used as transparent electrodes in almost all optoelectrical devices. InGaZnO, a representative transparent semiconductor, has been commercialized as a backplane transistor driving organic light‐emitting diode (OLED) displays.^[^ ^6^, ^7^, ^8^ ^]^ The ionic bonding feature and the high dispersion conduction band minimum (CBM) provide wide optical band gaps (*E*_g_ > 3.1 eV) and enable excellent electron transport, even in the amorphous structure. The low formation energy and shallow doping properties of the intrinsic donor defects (that is, oxygen vacancies) enable high and stable n‐type conductivity while maintaining optical transparency in visible light. Nevertheless, it is noted that almost all commercially available TS/C show n‐type conductivity, whereas high‐performance p‐type counterparts have not yet been developed.^[^ ^9^, ^10^, ^11^ ^]^ One reason is the limited variety of p‐type transparent TS/C as compared to those of n‐type oxides. Additionally, the valence band maximum (VBM) for hole transport in metal oxides is mainly comprised of anisotropic oxygen 2p orbitals. The small oxygen atom with high electronegativity makes the introduction of shallow acceptors difficult and the VBM is strongly localized, resulting in a low hole concentration, large hole effective mass, and low hole mobility. Another problem is that it is difficult to p‐dope the TS/C because of defect compensation.^[^ ^12^, ^13^ ^]^

Transparent electronics is one of the most important technologies for next‐generation information displays and the forecast shows that a $87.2 billion market would be created in transparent displays by 2025.^[^ ^14^ ^]^ Despite these great challenges, developing high‐performance p‐type TS/C is extremely important for many applications. For OLED and related optoelectronic devices, a p‐type transparent conductor (TC) with a high work function is more suitable than its n‐type counterpart because the highest occupied molecular orbital of the typical organic semiconductors is greater than 5.0 eV. Owing to the lack of good p‐type transparent electrode materials, current OLED and organic or perovskite solar cell devices require a hole injection and transport layer (HTL) to achieve efficient hole injection in ITO transparent electrodes.^[^ ^15^ ^]^ The water splitting process by sunlight also requires a robust transparent p‐type electrode for efficient hole collection.^[^ ^16^ ^]^ Furthermore, high‐performance p‐type TS/C is an essential building block for electrical contact and active layers, which can realize invisible p–n junction diode based optoelectronics and complementary metal oxide semiconductor (CMOS) circuits with low power consumption and high integration density by combination with n‐type metal oxides.

Pioneer research on p‐type transparent oxide conductors (TOCs) was reported in 1997. Kawazoe et al. proposed delafossite CuAlO_2_ based TOCs with high optical transparency (*E*_g_ = 3.5 eV) which encourages hole conductivity (1 S cm^−1^) by “chemical modulation of the valence band” approach.^[^ ^17^ ^]^ Since then, extensive studies have been conducted with two families of Cu(I)‐based TCs, including CuMO_2_ (M = Ga, Cr, In, Y, Fe, or others) delafossites and nondelafossites SrCu_2_O_2_ and LnCuOCh (Ln = La–Nd, Ch = S and Se).^[^ ^11^, ^18^, ^19^, ^20^, ^21^, ^22^ ^]^ Unfortunately, to date, it is still very difficult to achieve both high mobility and transparency even in the single‐crystalline form. Additionally, rigorous deposition techniques, such as pulsed laser deposition (PLD) and epitaxy growth, and high crystallization temperatures (≥700 °C) are not suitable for large‐area commercialization process. As for the p‐type semiconductors, only Cu_2_O, SnO, NiO, and Rh_2_O_3_ were demonstrated as potential candidates, but they suffered from insufficient hole transport with low mobility and inadequate optical transparency.^[^ ^9^, ^11^, ^12^ ^]^

The query remains open as to whether new high‐performance p‐type TC/S can be developed. The most recent computational prediction discovered the layered oxychalcogenide of [Cu_2_S_2_][Ba_3_Sc_2_O_5_] with a high *E*_g_ of 3.24 eV and p‐type conductivity exceeding 2000 S cm^−1^.^[^ ^23^ ^]^ Meanwhile, interest has grown regarding the search for p‐type excellent TC/S beyond oxides which can be processed by using simple and scalable methods at plastic compatible temperatures. Theoretical studies indicate that replacing O^2−^ with smaller electronegativity and larger p‐orbital anions could realize more delocalized valence bands (VB) than oxides.^[^ ^24^ ^]^ Following this strategy, metal chalcogenides and halides are promising alternatives. For metal chalcogenides, the Cu–Zn–S system is a typical example with both high conductivity of ≈3 S cm^−1^ and *E*_g_ of 3 eV.^[^ ^25^ ^]^ In addition to the more dispersed VB, as compared to the oxide materials, chalcogenides are easier to be p‐doped owing to their smaller ionization energies and higher‐lying p‐orbital derived VB.^[^ ^26^ ^]^ A recent review by Zakutayev and co‐workers systematically summarized the topic of “wide band gap (*E*_g_ > 2 eV) metal chalcogenides.”^[^ ^27^ ^]^ They highlighted that there are fewer stable chalcogenides than oxides, partially due to their decomposition tendency under ambient conditions, but it is interesting to explore the new chalcogenides. It is noted that many copper(I)‐based sulfide are degenerated semiconductors. The carrier polarity is almost nonsensical in degenerate semiconductors and the essence of semiconductor is the intentional controllability of Fermi level. Another candidate is the pseudohalide copper(I) thiocyanate (CuSCN), which shows a large *E*_g_ of 3.5 eV with decent hole transport due to the Cu 3d states dominated VBM, similar to other Cu(I)‐based oxides. High‐performance optoelectronic devices, such as solar cells, have been demonstrated using CuSCN as the hole transport layer.^[^ ^28^, ^29^, ^30^, ^31^, ^32^ ^]^ However, owing to the relatively large hole effective mass of 0.8 *m*_e_, CuSCN showed low p‐type conductivity with hole mobilities of <0.1 cm^2^ V^−1^ s^−1^, limiting its application in high‐performance electronic devices, e.g., transistor.^[^ ^33^ ^]^

**Figure 1:**
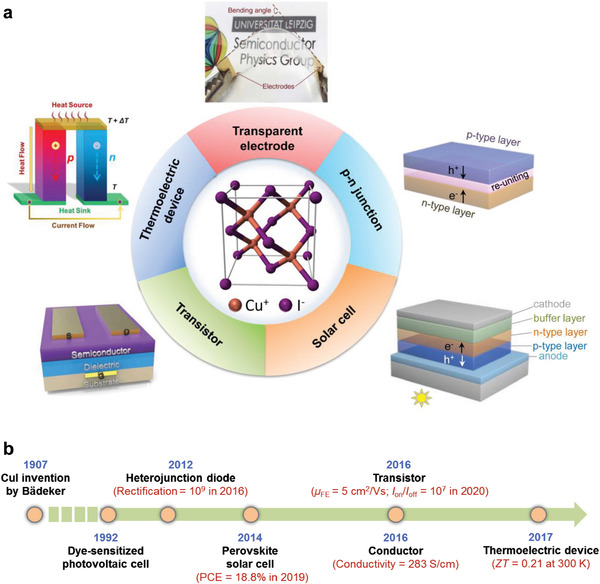
a) Schematic illustration of the CuI lattice structure and its diverse applications as a TC. Transparent electrode: Reproduced with permission.^[^ ^37^ ^]^ Copyright 2017, Nature Publishing Group. Transistor: Reproduced with permission.^[^ ^46^ ^]^ Copyright 2017, Wiley‐VCH. Thermoelectric generator: Reproduced with permission.^[^ ^2^ ^]^ Copyright 2020, American Chemical Society. b) Emerging applications achieved with CuI and the state‐of‐the‐art performance.

By contrast, transparent copper(I) iodide (CuI), with an *E*_g_ of 3.1 eV, shows a higher hole mobility, of up to 44 cm^2^ V^−1^ s^−1^, and a p‐type conductivity (maximum: 280 S cm^−1^) by appropriate doping with a wide range of hole concentrations (10^16^ to 10^20^ cm^−3^).^[^ ^34^, ^35^ ^]^ The superior electrical performance of CuI, as compared to Cu(I)‐based oxides, is beneficial from two aspects, namely, 1) the smaller electronegativity of iodine compared against that of oxygen (2.66 against 3.44 on the Pauling scale) enables more delocalized holes above the VBM^[^ ^36^ ^]^ and 2) the large I^−^ radius of 220 pm and the spatially spread three outermost p‐orbitals can achieve sufficient orbital overlap for fast hole transport. Additionally, CuI consists of abundant nontoxic elements and can be grown by a variety of film deposition methods at low temperatures (further detailed in the following section).^[^ ^37^, ^38^ ^]^ Owing to these features, CuI has been widely used in heterojunction diodes, thermoelectric devices, photodetectors, and as TCs and hole‐transport layers in photovoltaic devices and transistors (**Figure** **1**).^[^ ^35^, ^37^, ^38^, ^39^, ^40^, ^41^, ^42^, ^43^, ^44^, ^45^ ^]^

Given the rapid progress over the recent years, this article aims to provide a comprehensive understanding of the electronic properties of CuI‐based materials and the latest advances in related devices. For a detailed introduction to CuI and development history, we suggest another review paper reported in 2013 by Grundmann et al.^[^ ^34^ ^]^ Our paper begins with a brief introduction of the theoretical background and development of CuI and then reviews the various deposition methods and film properties. Subsequently, different doping/alloying approaches to modulate the optoelectrical properties of bare CuI are discussed. An overview of various device applications using CuI‐based TS/C follows. Finally, the current challenges and future perspectives of CuI for practical technology applications are presented.

## 2 CuI Introduction, Deposition Method, and Electrical Property Modulation

### 2.1 Basic Introduction of CuI

**Figure 2:**
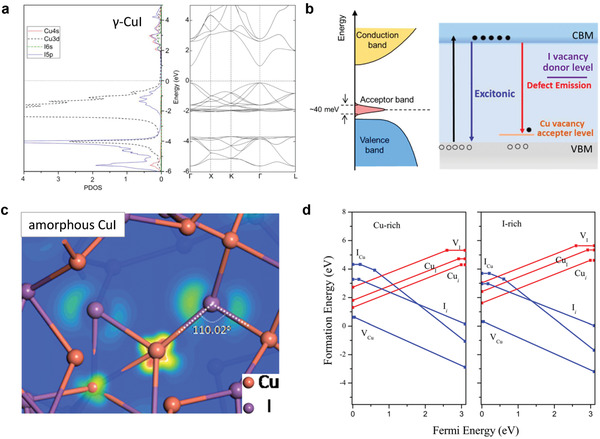
a) Projected density of state and corresponding band structure of *γ*‐CuI. Reproduced with permission.^[^ ^49^ ^]^ Copyright 2012, Institute of Physics. b) Band diagrams and schematic photoluminescence emission band structure of *γ*‐CuI with Cu acceptor states. Reproduced with permission.^[^ ^36^ ^]^ Copyright 2016, American Chemical Society. Reproduced with permission.^[^ ^72^ ^]^ Copyright 2019, American Chemical Society. c) Wave function of the VBM state in the amorphous CuI. Reproduced with permission.^[^ ^51^ ^]^ Copyright 2020, American Physical Society. d) Native defect formation energies calculated under the Cu‐/I‐rich condition. Reproduced with permission.^[^ ^50^ ^]^ Copyright 2011, American Institute of Physics.

In 1907, Bädeker reported the first TC of CuI by iodizing metallic Cu in iodine vapor.^[^ ^47^ ^]^ The obtained CuI thin films (200–300 nm) were fully transparent in the visible light range with a low resistivity of 4.5 × 10^–2^ Ω cm^−1^. The CuI showed a stable zincblende phase (*γ*‐CuI) below 350 °C, which is a typical p‐type semiconductor. The crystal structure transforms to wurtzite (*β*‐CuI) between 350 and 380 °C and rock salt (*α*‐CuI) at higher temperatures.^[^ ^48^ ^]^ The *β*‐ and, especially, *α*‐CuI are the conductor, which is mainly caused by the mobile Cu ions.^[^ ^34^ ^]^ In *γ*‐CuI, the VBM consists of Cu 3d and I 5p hybridization orbitals and the copper vacancy (*V*_Cu_) forms shallow acceptor states above the VBM (**Figure** **2**a,b).^[^ ^49^ ^]^ The lowest CBM is mainly made up of Cu‐s states. The large spatial spread of the I 5p orbitals enables sufficient overlap between adjacent I 5p orbitals intervening in a Cu 3d orbital, leading to a small hole effective mass of 0.3 *m*_e_ and a high mobility (**Table** **1**). The Cu 3d orbital plays a critical role in its electronic properties and the VB of CuI is derived from the filled d^10^ states in addition to the s^2^p^6^ shells.^[^ ^50^ ^]^ A recent theoretical investigation revealed that amorphous CuI has a similar VBM bonding state and hole effective mass to the crystalline phase (Figure 2c).^[^ ^51^, ^52^ ^]^ The network of amorphous CuI shows a tetrahedral structure and consists of two‐center bonds of Cu and I sp^3^ hybrids. The corresponding VBM is three neat‐degenerate states and this Cu–I–Cu unit is less sensitive to structural disorder. Among the native defects in *γ*‐CuI, *V*_Cu_ has the lowest ionization/formation energy in both Cu/I‐rich equilibrium growth conditions (Figure 2d). The intrinsically low formation energy of *V*_Cu_ in Cu(I)‐based semiconductors can be attributed to the antibonding character of VBM with high‐energy lying d^10^ orbitals.^[^ ^53^ ^]^ The antibonding interaction above the valence band tends to be tolerant to defects and the optoelectrical properties can be kept even with the existence of crystallographic defects.^[^ ^54^, ^55^ ^]^ The large iodine anion with the low coordination number of 2 further aids the defect tolerance ability.

**Table 1:** Carrier effective mass and bandgaps for p‐type oxide and (pseudo)halide semiconductors

Material	Structure	(*m*_h_) (Electron rest mass, *m*_e_)	Bandgap [eV]	Ref.
Cu_2_O	Cubic	*m*_h_ = 1.55 *m*_e_	≈2.20	^[^ ^58^ ^]^
SnO	Tetragonal	*m*_h_ = 2.05 *m*_e_	2.70 (direct) 0.70 (indirect)	^[^ ^59^, ^60^ ^]^
NiO	Cubic	*m*_h_ ≈ 1.00 *m*_e_	3.6	^[^ ^61^, ^62^ ^]^
ZnRh_2_O_4_	Spinel	*m*_h_ ≈ 3.50 *m*_e_	2.74	^[^ ^63^ ^]^
CuAlO_2_	Delafossite	*m*_h_ ≈ 2.60 *m*_e_	3.00	^[^ ^13^, ^64^ ^]^
CuCrO_2_	Delafossite	*m*_h_ ≈ 4.53 *m*_e_	3.20	^[^ ^65^ ^]^
SrCu_2_O_2_	Tetragonal	*m*_h_ ≈ 2.10 *m*_e_	3.30	^[^ ^66^ ^]^
La_2_SeO_2_	–	*m*_h_ = 0.92 *m*_e_	3.49	^[^ ^67^ ^]^
ZrOS	Tetragonal	*m*_h_ = 0.24 *m*_e_	2.5	^[^ ^68^ ^]^
CuAlS_2_	Zincblende	*m*_h_ = 0.33 *m*_e_	3.7	^[^ ^69^ ^]^
CsSnI_3_	Orthorhombic	*m*_h_ = 0.07 *m*_e_	1.3	^[^ ^70^ ^]^
CuSCN	Hexagonal	*m*_h_ = 0.80 *m*_e_ (*c‐*axis)	≈3.8	^[^ ^71^ ^]^
		*m*_h_ = 0.50 *m*_e_ (*ab‐*plane)		
CuI	Zincblende	*m*_h_ = 0.30 *m*_e_	3.1	^[^ ^34^ ^]^

The easy formation of *V*_Cu_ also makes the deposited *γ*‐CuI thin films highly conductive with high hole densities in the range of 10^17^–10^20^ cm^−3^.^[^ ^50^ ^]^ CuBr (*E*_g_ = 2.9 eV) and CuCl (*E*_g_ = 3.4 eV) also showed a zincblende structure with p‐type conductivity due to the facile formation of *V*_Cu_. However, because of the stronger bonding between Cu—Br and Cu—Cl than Cu—I, the hole concentration was significantly reduced from undoped CuI (>10^17^ cm^−3^) to CuBr (≈10^16^ cm^−3^) and CuCl (<10^13^ cm^−3^).^[^ ^56^ ^]^ Additionally, owing to the strong reducing ability of I^−^ and the large ionic radius difference between Cu^+^ and I^−^, Cu^+^ is the stable oxidization state in CuI. This is quite different from that of oxide‐based materials, where Cu^2+^ is favored.^[^ ^57^ ^]^ Another promising characteristic for CuI is its low‐temperature synthesis capability, which is not like other p‐type TC/S requiring strict deposition conditions at high temperatures. The stable cubic structure of *γ*‐CuI makes it easy to deposit using a variety of physical vapor and chemical solution methods, which are summarized in the following section.

### 2.2 CuI Thin Film Deposition Techniques

#### 2.2.1 Physical Vapor Deposition

**Figure 3:**
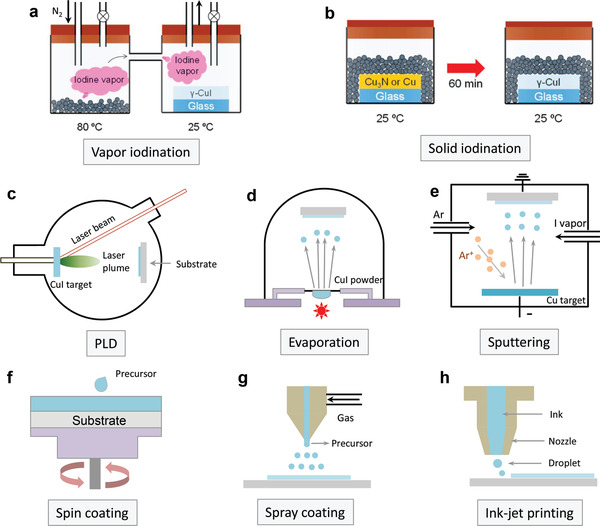
Schematic illustrations of a) vapor and b) solid iodination methods. Reproduced with permission.^[^ ^36^ ^]^ Copyright 2016, American Chemical Society. Different CuI thin film deposition technologies using c) PLD, d) thermal evaporation, e) reactive sputtering, f) spin‐coating, g) spray coating, and h) inkjet printing.

Since the first report using a Cu reaction with iodine vapor in 1907, this method has become the most common route for *γ*‐CuI film deposition.^[^ ^34^, ^41^, ^73^, ^74^, ^75^, ^76^, ^77^, ^78^ ^]^ The Cu film can be quickly converted into CuI after exposure to iodine vapor. Owing to the iodine volatility, the chemical reaction between Cu and iodine vapor is accelerated at a moderate temperature of 120 °C and the transparent *γ*‐CuI thin film can be obtained within 20 min (**Figure** **3**a). The iodization rate gradually decreases at temperatures above 150 °C. The deposited CuI thin films exhibit good p‐type semiconducting properties with a hole density of 5 × 10^18^ cm^−3^ and a hole mobility of 6 cm^2^ V^−1^ s^−1^. Unfortunately, owing to the fast chemical reaction, the CuI thin film exhibits a rough surface with a root‐mean‐square (RMS) roughness of >30 nm. Recently, Cao and co‐workers developed a solution iodination approach by dipping a Cu film in an iodine dissolved ethanol solution at room temperature (RT).^[^ ^79^ ^]^ The obtained CuI thin film delivered a high transparency of >70%, a low resistivity of 0.02 Ω cm, and a high Hall mobility of 34 cm^2^ V^−1^ s^−1^. Yamada et al. then developed an easy solid‐iodination method to deposit *γ*‐CuI through a chemical reaction between Cu_3_N and solid‐phase iodine at RT (Figure 3b).^[^ ^36^ ^]^ The obtained CuI thin films exhibited an optical transmittance of 70% at 500 nm, an improved surface morphology with an RMS ≈ 10 nm, and high Hall mobilities between 9 and 21 cm^2^ V^−1^ s^−1^. PLD‐fabricated CuI films were achieved by sintering the CuI ceramic target using a KrF excimer laser (Figure 3c).^[^ ^80^, ^81^, ^82^ ^]^ More recent studies have systematically investigated the growth and fundamental properties of PLD‐grown CuI thin films.^[^ ^82^ ^]^ With an increase in the growth temperature, the hole concentration decreased and the mobility increased owing to the reduced ionized impurity scattering. The hole concentrations could be modulated over a wide range of 5 × 10^16^–10^19^ cm^−3^ with the highest Hall mobility up to 20 cm^2^ V^−1^ s^−1^ at 240 °C. Similar to the iodization deposition route, the film surface was relatively rough (RMS ≈ 4 nm) and smooth surfaces were only available at low growth temperatures. Another shortcoming of PLD technique is the low deposition efficiency and high cost, limiting large‐area mass fabrication.

By using thermal evaporation (Figure 3d), Grundmann et al. deposited a transparent CuI thin film with a low RMS value of 2 nm at RT.^[^ ^34^ ^]^ An enlarged grain size was observed at elevated annealing temperatures.^[^ ^83^, ^84^ ^]^ The highest Hall mobility of 25 cm^2^ V^−1^ s^−1^ and conductivity of 100 S cm^−1^ were reported by Cao and co‐workers under the optimized substrate temperature of 120 °C.^[^ ^83^ ^]^ For the sputtering technique, nonreactive radio frequency (RF), direct current (DC), and magnetron sputtering proved feasible for depositing CuI thin films.^[^ ^35^, ^37^, ^43^, ^85^, ^86^, ^87^, ^88^ ^]^ In comparison, reactive sputtering has intrinsic advantages over conventional RF and DC sputtering when considering the difficulties in sintering the CuI target. Here, a metallic Cu disk was used as the DC sputtering source with continuous‐input iodine vapor (Figure 3e). Benefiting from the high conductivity of the Cu target, high film uniformity could be achieved owing to the high plasma density. Using this method, the Cu/I ratio could be easily modulated and the highest CuI conductivity of 283 S cm^−1^ was achieved with I‐rich CuI.^[^ ^35^ ^]^ Nevertheless, the only scanning electron microscope image showed an uneven film surface without film roughness details.^[^ ^43^ ^]^ Most recently, Yang and colleagues systematically investigated the growth mechanism and kinetics of sputtered CuI thin films.^[^ ^87^ ^]^ The interface properties between adatom/substrate driven by the deposition temperature and growth rate played important roles in forming uniform CuI samples and the smooth films with RMS values of 2–3 nm were achieved at a high substrate temperature of 358 °C with a high growth rate. In addition to the above commonly used techniques, *γ*‐CuI thin films were successfully deposited by electrodeposition,^[^ ^89^ ^]^ glancing angle deposition,^[^ ^90^ ^]^ hydrothermal growth,^[^ ^91^ ^]^ and molecular beam epitaxy.^[^ ^92^ ^]^ Among these physical vapor deposition techniques, thermal evaporation is one very promising choice, which not only possesses the simplicity and high reproducibility by using commercialized CuI powder, but also enables the growth of high‐quality and smooth CuI thin films over large area at plastic‐compatible temperatures.

#### 2.2.2 Chemical Solution Process

The continuous pursuit of cost‐effective manufacturing has refocused the research efforts to the solution‐based coating and printing approaches because they do not require expensive high‐vacuum processing chambers.^[^ ^93^ ^]^ Additionally, the solution process has other advantages, such as roll‐to‐roll/large‐area/atmospheric deposition capabilities, easy operation, and component adjustment. More importantly, unlike solution‐processed metal oxide thin films which require relatively high annealing temperatures (≥300 °C) for precursor conversion and film densification, the CuI film can be directly deposited from solution at relatively low temperatures (<150 °C).^[^ ^9^, ^44^, ^94^, ^95^, ^96^ ^]^ Benefiting from its simplicity and low cost, spin‐coating has become the most commonly used solution‐processing method in laboratories (Figure 3f). To prepare the precursor inks, the CuI powder can be easily dissolved in different solvents, such as acetonitrile,^[^ ^40^, ^44^, ^97^, ^98^, ^99^ ^]^ 2‐methoxyethanol,^[^ ^42^, ^100^ ^]^ dipropyl sulfide,^[^ ^101^, ^102^ ^]^ and even deionized water.^[^ ^103^ ^]^ Solvent selection has a significant effect on the CuI film deposition temperature and quality. When 2‐methoxyethanol (boiling point: 124 °C) was used, moderate baking at ≈130 °C was required to evaporate the residual solvent. By contrast, the adoption of acetonitrile with a low boiling point of 82 °C enables lower temperature, or even RT, deposition. Although the postannealing process can enhance film crystallinity with larger grains, thermal heating can also cause film aggregation and evaporate the lattice iodine from CuI owing to the low lattice formation energy.^[^ ^44^ ^]^ The iodine vacancy (*V*_i_) acts as a trap state and hole scatters, which can compensate the acceptor, reduce the hole density, and degrade the hole transport property accordingly. Additionally, solvent selection has a significant effect on the film surface morphology, and the use of acetonitrile was beneficial for growing smooth CuI thin films with small RMS values of <1 nm. Our group recently conducted systematic investigations on spin‐coated CuI thin films and demonstrated their application potential in thin‐film transistors (TFTs).^[^ ^44^ ^]^ The as‐coated CuI thin films exhibited a polycrystalline structure with a high optical transmittance of >90%, a smooth surface with an RMS value of 0.6 nm, and a Hall mobility of 5.1 cm^2^ V^−1^ s^−1^. As the annealing temperature increased, the film crystallinity was dramatically enhanced with accelerated iodine decomposition at >60 °C and the CuO phase appeared after annealing at 200 °C in air. By using more scalable techniques, CuI thin films were deposited using spray coating^[^ ^104^ ^]^ and ink‐jet printing (Figure 3g,h).^[^ ^105^ ^]^ Generally, each deposition approach has its unique advantages and intrinsic limitations. Taking most commonly used spin‐coating method for instance, despite the good reproducibility and simplicity, it limits in the scalability for large‐area deposition and most of the solution (95%) is wasted during the film deposition.

To fabricate high‐quality CuI thin films by a solution process, solvent selection and film coating/annealing approaches should be carefully considered. The solvent characteristics, e.g., viscosity, toxicity, boiling point, polarity, and coordination ability, play key roles in determining the film forming dynamics and thus the film quality no matter which solution deposition method is employed. Currently, the low‐toxic acetonitrile is the most popular solvent used for solution‐processing of CuI thin films. Its low boiling point leads to the fast volatility during the coating process and thus the poor film crystallinity and uniformity. To adjust the evaporation rate of solvent, besides the boiling point and viscosity, the solute–solvent coordination interaction should be noted.^[^ ^28^ ^]^ The soft acid character of Cu^+^ tends to form strong coordination complexes with the solvent containing S‐ and N‐ ligands.^[^ ^106^ ^]^ To reduce the possible solvent residues in low‐temperature‐annealed CuI thin films, the antisolvent treatment can be employed, which can also improve the film crystallinity.^[^ ^107^ ^]^ In fact, the solvent engineering has been largely overlooked and the further efforts on the low‐toxic solvent screening are very interesting and worthy to explore. Similar to the solvent selection, the commonly used deposition and post‐treatment approaches are unitary, i.e., spin‐coating and thermal annealing. The rapid spin during the film coating process leads to the fast and uncontrollable solvent evaporation, which causes the fast crystallization with the aggregation of CuI cluster. The development of alternative solution deposition method is interesting to grow high‐quality CuI film over large area with smooth surface and great uniformity. One promising option is the blade/bar coating, which has been demonstrated effectively in improving crystallinity and film quality of organic, metal oxide and halide perovskite semiconductors.^[^ ^108^, ^109^, ^110^ ^]^ Considering the low formation energy of CuI, we suggest the novel photonic processing techniques to reduce the postannealing temperature and time relative to conventional thermal heating.^[^ ^93^, ^111^ ^]^ The light sources, such as deep ultraviolet, laser, flash lamp annealing, can deliver precise interaction between photons and CuI layer and provide energy within µs to ms time range.

### 2.3 Electrical Property Modulation of CuI via Alloying/Doping

**Figure 4:**
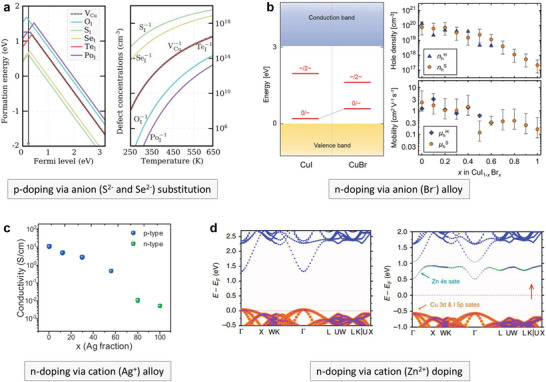
a) Formation energies of native *V*_Cu_ and indicated p‐type dopants under Cu‐rich conditions; right panel describes the thermal equilibrium charged defect concentrations as a function of temperature. Reproduced with permission.^[^ ^120^ ^]^ Copyright 2019, Royal Society of Chemistry. b) Charge‐state transition levels of *V*_Cu_ in CuI and CuBr (left). The hole concentrations and mobilities of CuI_1‐_ *_x_* Br*_x_* thin films determined from Hall‐effect and Seebeck‐effect measurements (right). Reproduced with permission.^[^ ^121^ ^]^ Copyright 2018, Wiley‐VCH. c) Conductivity variations for Ag*_x_* Cu_1‐_ *_x_* I alloys as a function of the Ag^+^ content. Reproduced with permission.^[^ ^78^ ^]^ Copyright 2020, Elsevier. d) Band structures of CuI and Zn^2+^‐doped CuI. Reproduced with permission.^[^ ^45^ ^]^ Copyright 2020, Nature Publishing Group.

**Table** **2** clearly shows that the physical vapor routes (such as PLD, sputtering, and evaporation) tend to grow high‐conductivity CuI for TCs, which possibly benefit from the high film purity and dense structure. Film deposition techniques using sputtering and evaporation are preferred for industrial mass production, while PLD and molecular beam epitaxy approaches are only suitable for laboratory research because of their limited film growth scale. In contrast, CuI thin films synthesized from a precursor solution generally contain a certain amount of impurities with a loose film density and therefore exhibit insufficient electrical conductivity. This offers the opportunity for semiconductor applications. Another advantage of the solution process is the easy incorporation of foreign atoms, which is convenient and efficient for investigating the doping/alloying effect. *γ*‐CuI is a well‐known transparent p‐type conductor owing to its relatively high conductivity and hole concentration. Additionally, a good p‐type TC should have doping capacity. Based on the established doping limit rule, CuI can be p‐doped with holes because its VBM level is shallower than −6 eV.^[^ ^50^, ^112^, ^113^ ^]^ For metal oxide materials, the easy formation of oxygen vacancies when lowering the Fermi level toward the VB has been identified as intrinsic compensation defects (hole killers) and has become the main impediment to p doping in transparent oxides.^[^ ^13^ ^]^ In addition to the transparent electrode application, high conductivity is beneficial for thermoelectric devices and as an HTL for photovoltaics. Considering the easy generation of *V*_i_ during the film coating/annealing processes due to its low formation energy, thiocyanate (SCN^−^) was blended into CuI to stabilize the lattice iodine and form smooth and compact composite films.^[^ ^114^, ^115^, ^116^ ^]^ By creating an I‐rich environment during reactive sputtering, the CuI film conductivity increased from 156 to 283 S cm^−1^.^[^ ^102^ ^]^ The same phenomenon was observed using the solution process by directly adding iodine solution into the CuI precursor, leading to a conductivity enhancement of more than two orders of magnitude.^[^ ^117^ ^]^ Later, a humidity‐induced conductivity increment was observed on CuI after exposure to moist air (relative humidity = 35%) for several hours.^[^ ^118^ ^]^ The reason was speculated as grain boundary passivation and nonporous filling by water in the molecular or dissociated form, thus improving the hole transport across grain boundaries. However, the increased conductivity brought by O_2_ absorption should be also included due to its electron scavenger effect.^[^ ^119^ ^]^ Recently, Flores‐Livas and co‐workers employed a high‐throughput computational approach to search for possible substitutional dopants for *γ*‐CuI.^[^ ^120^ ^]^ The results showed that chalcogen elements had an acceptor character and one hole could be released after replacing lattice iodine. In particular, sulfur and selenium substitution can increase the hole concentration (**Figure** **4**a).

**Table 2:** Opto/electrical parameters of CuI thin films deposited using different methods

Method	Hall mobility [cm^2^ V^−1^ s^−1^]	Hole concentration [cm^−3^]	Growth temp. [°C]	Transmittance at 500 nm	Conductivity [S cm^−1^]	RMS [nm]
Solid iodination	9–21	4 × 10^17^–10^18^	120–150	≈70%	≈1	8–12
Solution iodination	34	10^18^–10^19^	RT	60%	50	≈30
Vapor iodination	6	10^18^–10^19^	120	50%	5	30–80
PLD	8–20	5 × 10^16^–10^19^	70–240	≈80%	100	1–4
Sputtering	3–9	10^19^–10^20^	RT	>60%	156–283	2–3
Evaporation	2–25	10^19^–10^20^	RT ≈ 200	>60%	100	2–5
Molecular beam epitaxy	≈10	≈10^18^	240	–	0.01–1	–
Spin‐coating	0.5–7	7 × 10^16^–5 × 10^19^	RT = 150	>70%	0.025–3.5	0.6–20
Ink‐jet printing	10.5	2.2 × 10^16^	60	>80%	–	3.24

In addition to the strategies to enhance the *γ*‐CuI conductivity, more efforts have been made recently to weaken the conductivity for use as transparent semiconductors because high hole concentrations impede the potential applications in electronic devices, such as diodes and transistors. The most direct way to suppress the hole number is heat treatment to generate more *V*_i_ (electron donor).^[^ ^44^ ^]^ Nevertheless, severer postannealing can cause film aggregation with undesired defects. Two effective hole‐suppression approaches by alloying and doping have been established. In 2020, Yamada et al. proposed a Br^−^ alloying route by thermally evaporating CuI and CuBr powders with different Br^–^ ratios.^[^ ^121^ ^]^ Poor p‐type semiconducting properties with low hole mobilities of 0.15–3 cm^2^ V^−1^ s^−1^ were demonstrated in the CuBr films and the resistivity was three orders of magnitude higher than that of CuI.^[^ ^122^, ^123^ ^]^ The theoretical calculation indicated a deeper CuBr *V*_Cu_ acceptor level than that in CuI (Figure 4b). Consequently, Br^−^ alloying greatly reduced the high hole concentrations from 10^20^ to 10^17^ cm^−3^. The polycrystalline CuI_1−_ *_x_* Br*_x_* alloy films displayed similar surface morphologies with RMS values in the range of 2–5 nm. Meanwhile, Br^−^ alloying had little effect on transparency and all alloys exhibited large *E*_g_ ≥ 3.0 eV. During the same period, Annadi and Gong reported another cation‐alloying approach using Ag^+^.^[^ ^78^ ^]^ The authors first used RF sputtering to deposit Ag*_x_* Cu_1‐_ *_x_* metal films and then reacted them with I_2_ vapor to obtain the Ag*_x_* Cu_1‐_ *_x_* I thin films. The neat CuI sample showed a high conductivity of 10.5 S cm^−1^, a hole density of 2 × 10^19^ cm^−3^, and a Hall mobility of 3.5 cm^2^ V^−1^ s^−1^. The 0.3 at% Ag^+^ alloy delivered the highest mobility of 8.1 cm^2^ V^−1^ s^−1^ with a reduced conductivity of 2.6 S cm^−1^ and a hole density of 2.2 × 10^18^ cm^−3^. Interestingly, when the Ag^+^ alloy ratio exceeded 0.8, the samples displayed electron‐dominating (n‐type) properties with a high electron mobility of 20.2 cm^2^ V^−1^ s^−1^ and an n‐type conductivity of 0.01 S cm^−1^ (Figure 4c). Ultraviolet photoelectron spectroscopy analysis confirmed the Hall measurement data. The Fermi level gradually shifted from VBM toward CBM as the Ag^+^ fraction increased. The Ag^+^ alloy also reduced the *E*_g_ from 3.02 (CuI) to 2.78 eV (AgI). Based on this study, it will be interesting to clarify the origin of enhanced electron transport with Ag^+^ alloy. In addition, the ion movement brought by AgI addition should be considered because AgI is a well‐known super ionic conductor.

Substitutional doping has been widely used to adjust the carrier concentration and conductivity of inorganic semiconductors. To achieve efficient substitutional n‐doping in CuI without distorting the host lattice, the ionic size of the dopant cation should be similar to that of Cu^+^ (77 pm). Meanwhile, the coordination number and geometry of the dopant cations should be the same as that of Cu^+^ to maintain the local lattice structure. Theoretical calculations compared CuI doping with Zn^2+^, Ga^3+^, and Al^3+^ and highlighted that the valence state difference between the Cu^+^ and dopant cations played an important role in predicting the dopant solubility in the lattice.^[^ ^124^ ^]^ In brief, the local distortions are large for Ga^3+^ and Al^3+^ because their effective charge is −2 in the lattice. To date, several dopant cations, that is, Ni^2+^ (69 pm), Sn^4+^ (71 pm), and Zn^2+^ (74 pm), have been incorporated into CuI through diverse deposition processes.^[^ ^42^, ^45^, ^97^, ^125^ ^]^ The hole concentration could be effectively reduced after Cu^+^ substitution. The addition of a suitable amount of foreign cations (<10 mol%) could also inhibit the fast crystallization of CuI thin films, achieving smooth surface morphologies. Among the different dopants, Zn^2+^ is one of the superior candidates because of its suitable ionic size, the same tetrahedral coordination structure, and the low formation energy of Zn_Cu_ (Figure 4d).

**Figure 5:**
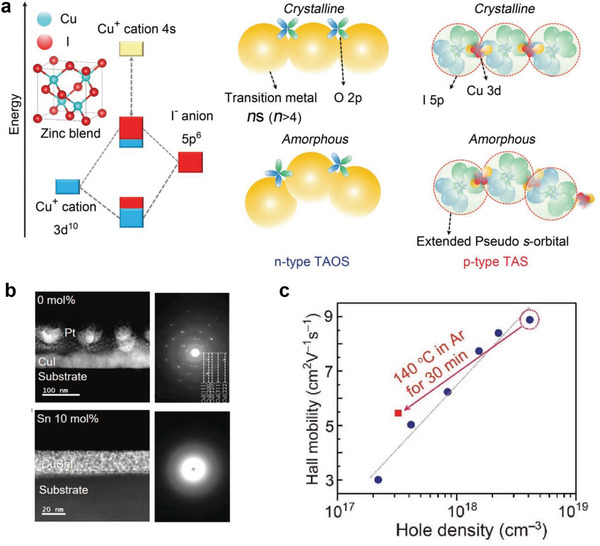
a) Illustration of the amorphous CuI design concept compared with the n‐type transparent amorphous oxide semiconductors. b) Transmission electron microscopy images and selected area electron diffraction patterns of CuI (upper) and 10 mol% Sn^4+^‐doped CuI thin films; and c) Hall mobility dependence on hole concentration for amorphous CuSnI. Reproduced with permission.^[^ ^42^ ^]^ Copyright 2018, Wiley‐VCH.

It is worth noting that the majority of reported transparent oxides and halide TS/Cs are polycrystalline and only a few components showed an amorphous nature with p‐type conductivity. The first amorphous p‐type oxide of ZnRhO*_x_* was reported by Narushima et al. in 2003.^[^ ^126^ ^]^ Later, V*_x_* O*_y_*,^[^ ^127^ ^]^ and BiRuO,^[^ ^128^ ^]^ and ZnRhCuO^[^ ^129^ ^]^ were synthesized but the hole mobilities were lower than 1 cm^2^ V^−1^ s^−1^. The polycrystalline films generally exhibited higher electrical performance as compared to the amorphous ones due to the locally enhanced orientation and reduced trap states, but meanwhile, a certain amount of grain boundaries can limit the large‐area uniformity. Hosono and co‐workers found that doping CuI with 5 mol% Sn^4+^ not only functions as an electron donor but also transforms the polycrystalline structure into an amorphous state (**Figure** **5**).^[^ ^42^ ^]^ Benefiting from the superior hole transport property in amorphous CuI, the amorphous 5 mol% Sn^4+^‐doped CuI thin film delivered a reasonable Hall mobility of ≈4 cm^2^ V^−1^ s^−1^ and a hole density of 2 × 10^17^ cm^−3^. The Hall mobility showed a positive correlation with the hole concentration (Figure 5c), implying the percolation dominated hole transport property. It should be noted that one following study revealed the polycrystalline texture of CuI even with the addition of 10 mol% Sn^4+^.^[^ ^97^ ^]^ This raises the question that whether the experimental details, such as the solvent selection and annealing conditions, have great effect on the film crystallinity. In addition, considering the poor stability of CuI in air, especially in humid atmosphere, the structural characterization should be carefully carried out. We suggest coating hydrophobic Cytop layer on the top of CuI sample to ensure the durability.

## 3 CuI Applications in Thermo/Optoelectronic Devices

### 3.1 Transparent Conductor

**Figure 6:**
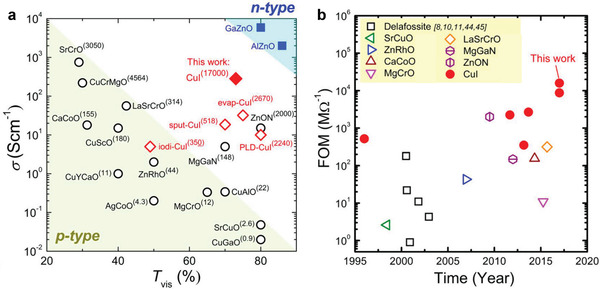
a) Summarization of RT electrical conductivity and averaged visible transmittances for CuI and other n‐ and p‐type TCs and b) comparison of FOM over time for the reported diverse p‐type TCs. Reproduced with permission.^[^ ^35^ ^]^ Copyright 2016, PNAS.

As previously mentioned, *γ*‐CuI was initially expected to be used as a transparent p‐type conductor. Considering the *V*_Cu_ dominated p‐type conductivity, the CuI growth under iodine‐rich conditions is beneficial for achieving high conductivity. Yang and co‐workers observed increased conductivity with controlled iodine partial pressure during sputtering and reported the highest p‐type conductivity of 283 S cm^−1^ (**Figure** **6**).^[^ ^35^ ^]^ The conductivity was stable in air and slightly degraded after 3 months of storage. To quantitatively evaluate the performance of the deposited CuI, the authors calculated the figure of merit (FOM) using the ratio of conductivity (*σ*) to the visible absorption coefficient (*α*). The value of *α* was computed from the total visible transmission and corrected for reflectance. In a neglecting reflectance mode, the FOM can be simplified to FOM ≈ −1/(*R*_s_ln*T*), where *R*_s_ is the sheet resistance. As shown in Figure 6b, the CuI exhibited a considerably higher FOM (1.3 × 10^6^ MΩ^−1^) than previously reported p‐type TCs and was even close to the standard ITO (4 × 10^6^ MΩ^−1^).^[^ ^65^ ^]^ Later, Raj et al. proposed another CuI/insulator composite conductor system by incorporating TiO_2_ into CuI.^[^ ^43^ ^]^ The results showed that the addition of TiO_2_ not only increased the transparency and hole conductivity by one order of magnitude, but also significantly improved the ambient stability of more than 6 months. The metallic Cu and TiO_2_ mixture was first deposited using a cosputtering system and then was exposed to the solid/vapor iodine to form CuI–TiO_2_ to further increase the conductivity. The segregation of TiO_2_ at the CuI grain boundaries enhanced the conductivity at the grain boundary interface. Meanwhile, the presence of TiO_2_ inhibited recrystallization and grain growth, CuI oxidation, and reduced iodine loss.

Although CuI exhibits considerably higher p‐type conductivity than p‐type metal oxides, it needs further improvement to replace n‐type TCOs. According to the formula *σ* = *enμ*, where *e* is the elementary charge, the electrical conductivity (*σ*) is directly related to its carrier concentration (*n*) and carrier mobility (*μ*). For industry‐standard ITO, an electron concentration of 10^21^ cm^−3^ and an electron mobility of 50 cm^2^ V^−1^ s^−1^ can be achieved, delivering a high conductivity of 10^4^ S cm^−1^. The currently reported conductive CuI thin films exhibit hole concentrations of ≈10^20^ cm^−3^ and hole mobilities in the range of 3.5 to 9 cm^2^ V^−1^ s^−1^. *n* is associated with the intrinsic ease of generation of mobile carriers by defects or dopants. As previously discussed, the I‐rich deposition condition and chalcogen elements (such as S^2−^ and Se^2−^) substitution are feasible approaches for increasing *n*. After I^−^ substitution, the extra holes can be easily activated into the VBM as free holes if the dopant energy levels are located above those of the VBM, leading to a significantly increased *σ*. According to *μ* = *eτ*/*m* ^*^, *μ* is directly proportional to the carrier scattering time (*τ*) and is inversely determined by the carrier effective mass (*m* ^*^). *m* ^*^ is an intrinsic parameter, while *τ* can be modified through extrinsic factors, such as ionized dopants, defects, and grain boundaries, by tuning the film deposition process. In this case, the approaches to enlarging the grain size or adopting the epitaxial technique to grow high‐quality CuI films are feasible to increase *τ* and enhance the hole mobility. Unfortunately, considering the highest hole mobility of 44 cm^2^ V^−1^ s^−1^ in a single CuI crystal, the mobility improvement seems limited.

### 3.2 Thermoelectric Devices

Over the past decade, research on thermoelectrics has achieved enormous development for energy conversion applications by identifying many new materials.^[^ ^3^ ^]^ To achieve high thermoelectric performance, the material should be electrically conductive and possess a large Seebeck coefficient with low thermal conductivity, as indicated by $ZT\;=\frac{S^{2}\sigma }{k}\;T$, where *S* is the Seebeck coefficient, *σ* is the electrical conductivity, *к* is the thermal conductivity, and *T* is the absolute temperature. Nevertheless, it is extremely difficult to control these parameters simultaneously because they are coupled to each other and attempts to improve one parameter could sacrifice the others.^[^ ^130^ ^]^ Additionally, the majority of commonly used thermoelectric materials are composed of toxic and expensive elements.^[^ ^2^ ^]^ Great efforts have been made to find new thermoelectric materials containing inexpensive and earth‐abundant elements for large‐area fabrication. Among these candidates, CuI is one of the most promising transparent candidates with high thermoelectric performance. The heavy iodine element and strong phonon scattering lead to a low thermal conductivity and a large Seebeck coefficient of 237 µV K^−1^ was theoretically predicted for *γ*‐CuI.^[^ ^131^ ^]^ More importantly, like complementary electronics, both n‐ and p‐type transparent thermoelectric materials are needed because the thermoelectric module involves coupled n‐/p‐type thermoelectric legs. However, as previously discussed, there is still no highly conductive p‐type TS/C.

**Figure 7:**
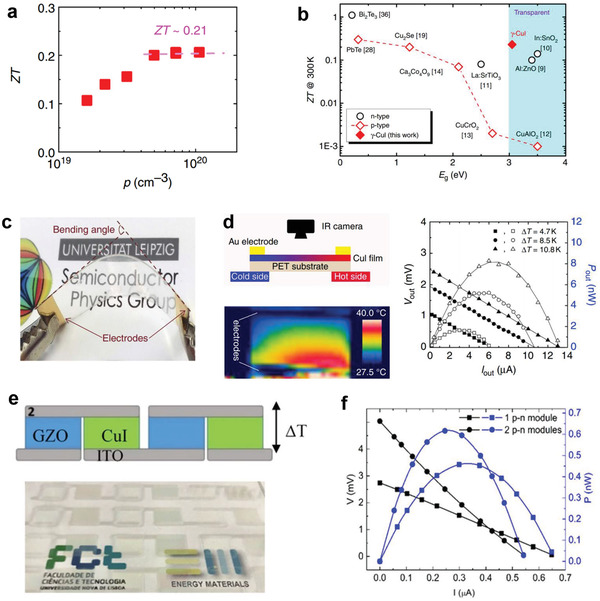
a) The variation of *ZT* values for the *γ*‐CuI thin films as a function of the hole density at RT. b) Comparison of *ZT* values and *E*_g_ for the typical n‐/p‐type thermoelectric materials at RT and c) the actual picture and d) output voltage/output power of the flexible CuI‐based single‐leg thermoelectric module. Reproduced with permission.^[^ ^37^ ^]^ Copyright 2017, Nature Publishing Group. e) Photograph and f) the thermoelectric properties of CuI/GaZnO p–n modules. Reproduced with permission.^[^ ^132^ ^]^ Copyright 2018, Nature Publishing Group.

A preliminary study was reported in 2017 by Yang et al. who grew 300 nm CuI thin films by reactive sputtering at RT.^[^ ^37^ ^]^ A large thermoelectric FOM of *ZT* = 0.21 at 300 K was achieved (**Figure** **7**a). It is worth noting that the high *ZT* value is 1000 times higher than that of p‐type TCOs and ≈100 times higher than that of n‐type TCOs (Figure 7b). Thereafter, the authors demonstrated a prototype of a flexible and transparent CuI‐based thermoelectric module with a high‐power density of 2.4 mW cm^−2^ at Δ*T* = 50 K (Figure 7c,d). In a follow‐up study, Faustino et al. combined solid iodinated CuI with n‐type Ga‐doped ZnO thin films to construct invisible and flexible p–n thermoelectric modules (Figure 7e,f).^[^ ^132^, ^133^ ^]^ The generated power output compared to the well‐known thermoelectric bulk component (Sb_2_Te_3_/Bi_2_Te_3_, *ZT* value ≈1).^[^ ^134^, ^135^ ^]^ These studies not only demonstrated the novelty of CuI as a transparent thermoelectric material, but also accelerated the realization of thermoelectric windows, body‐heat‐driven wearable electronics, and on‐chip cooling or power reversion for miniaturized chips. To further enhance the thermoelectric performance of CuI, the film conductivity should be improved. In addition, due to the low formation energy of CuI lattice (common issue for metal halides), the iodine loss under long‐term thermal environment is inevitable. Therefore, the feasibility of metal‐halide materials as potential thermoelectric components should be carefully evaluated. The sulfur and selenium substitution seems an promising approach, which can not only increase the thermal stability but also the hole concentration.

### 3.3 Heterojunctions for Diode, Photodetector, LED, and Piezoelectric Enhancement

**Figure 8:**
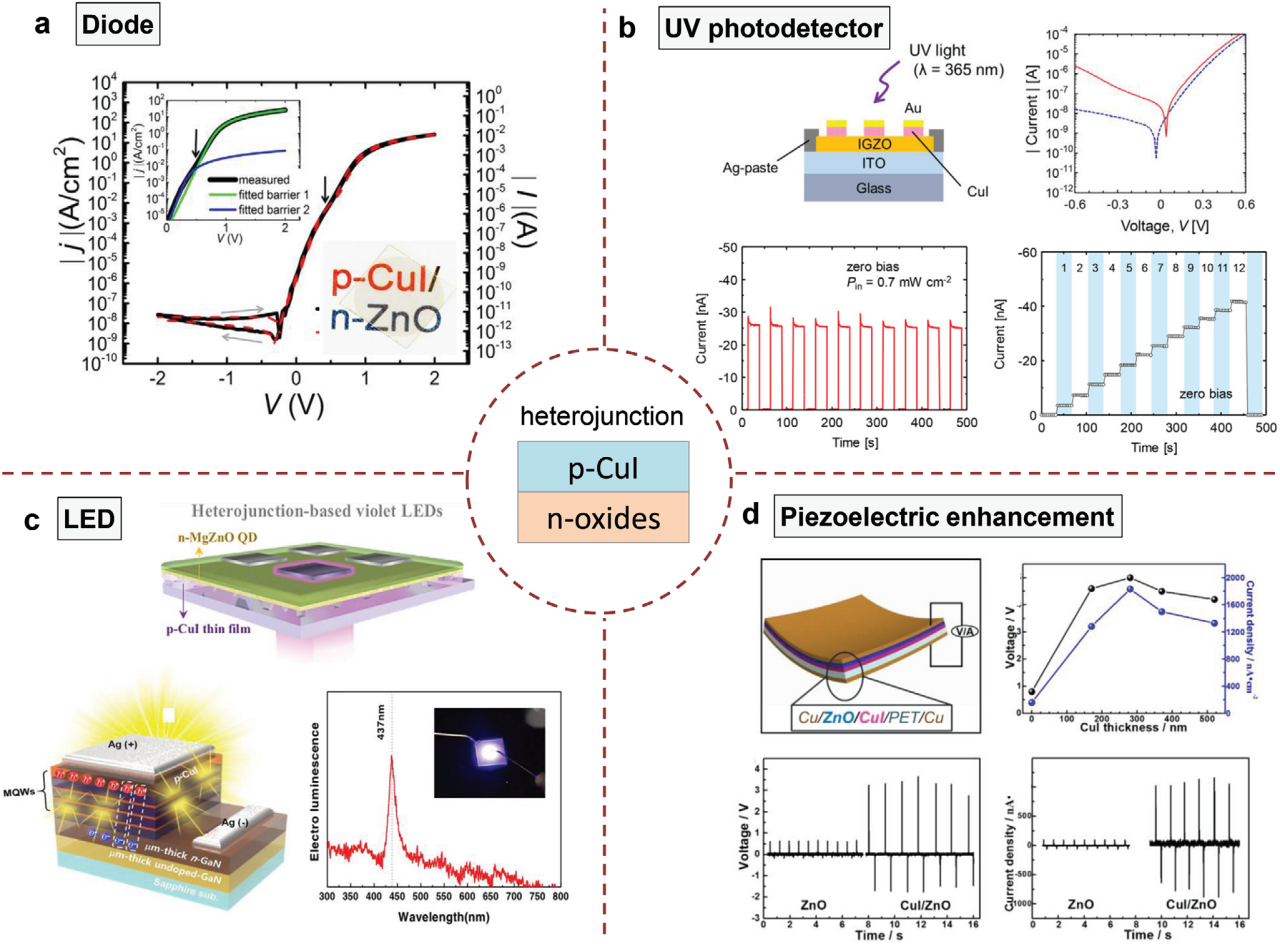
a) Current density against voltage curves of a p‐CuI/n‐ZnO diode. The inset depicts the device photograph. Reproduced with permission.^[^ ^139^ ^]^ Copyright 2016, Nature Publishing Group. b) Photodetector structure, current–voltage curves, photoresponse test, and photocurrent under different UV intensities of a CuI/IGZO heterojunction. Reproduced with permission.^[^ ^141^ ^]^ Copyright 2019, Elsevier. c) LED structure and electroluminescence spectrum. Bottom images: Reproduced with permission.^[^ ^145^ ^]^ Copyright 2020, Nature Publishing Group. Top image: Reproduced with permission.^[^ ^146^ ^]^ Copyright 2020, American Chemical Society. d) The structure of a flexible CuI/ZnO piezoelectric nanogenerator and the device output performance with different CuI thicknesses. Reproduced with permission.^[^ ^77^ ^]^ Copyright 2016, Elsevier.

The p–n junctions are fundamental building blocks for optoelectronic devices. CuI thin films showed great compatibility with various n‐type metal oxide/halide semiconductors (such as, ZnO, BaSnO_3_, a‐IGZO, AgI, and NiI_2_) to construct transparent p–n hetero/homojunction diodes.^[^ ^38^, ^41^, ^125^, ^136^, ^137^, ^138^, ^139^ ^]^ Grundmann and co‐workers fabricated an epitaxial thin‐film heterojunction of p‐CuI/n‐ZnO with a high rectification of up to 2 × 10^9^ and a low saturation current density of 5 × 10^−9^ A cm^−2^ (**Figure** **8**a).^[^ ^139^ ^]^ The rectification ratio was ≈100 times higher than that of polycrystalline CuI‐based diodes. This inspires us the future efforts should be paid on high‐quality CuI growth for boosting the device performance no matter which deposition method is adopted. Later, Yamada et al. observed the photovoltaic effect in a CuI/IGZO heterojunction under weak UV illumination.^[^ ^140^ ^]^ This finding has triggered its potential application as a UV photodetector.^[^ ^97^, ^141^, ^142^ ^]^ CuI could absorb UV light owing to the simultaneous band‐to‐band and excitonic absorptions at RT. Initial studies with core/shell‐ and planar‐type CuI/ZnO UV photodetectors exhibited excellent UV‐detection properties owing to the formation of a built‐in electric field and the combination with a piezoelectric polarization field.^[^ ^77^, ^142^ ^]^ Recently, a fast‐response self‐powered UV photodetector was reported using the RT‐fabricated CuI/IGZO heterojunction diode (Figure 8b).^[^ ^141^ ^]^ The device showed sufficient detectivity to zero‐bias photocurrent upon UV irradiation and therefore could serve as a self‐power operated photodetector. Meanwhile, the photocurrent showed a fast response (rise time: 2.5 ms and decay time: 35 ms) to the ON/OFF states of UV light and great linearity with the UV light intensity. The response speed was faster than those of self‐powered UV photodetectors using nitride and wide bandgap oxide semiconductors. During the same period, Zhang et al. extended the light sensing range to visible light by combining CsPbBr_3_ perovskite with CuI and reported a self‐powered visible light photodetector with excellent detection properties.^[^ ^143^ ^]^

Benefiting from the large exciton binding energy of CuI, that is, 58 meV, CuI has recently been employed in highly efficient blue and UV LEDs and is expected to replace commercialized GaN in the future.^[^ ^144^, ^145^, ^146^ ^]^ In 2020, Ahn et al. grew epitaxial p‐CuI as a hole injection layer and reported a hybrid n‐GaN/p‐CuI blue LED.^[^ ^145^ ^]^ The epitaxial CuI showed superior electrical properties as compared to the early developed p‐GaN for commercial LEDs. A stronger photoluminescence peak intensity was also observed in the CuI film. Figure 8c displays the LED structure and the electroluminescence spectrum (peak at 437 nm) of the device. However, the corresponding quantum efficiency is missing in this work. In the same year, violet LEDs based on p‐CuI/n‐Mg*_x_* Zn_1−_ *_x_* O quantum dot heterojunctions were reported.^[^ ^146^ ^]^ The CuI layer was prepared using the vapor iodination method and the processing temperature played a crucial role in the morphology, optical, and electrical properties of the obtained CuI layer. The optimized iodination temperature of 15 °C achieved a high hole concentration in the p‐CuI thin film, leading to an improvement in the LED performance with a strong violet emission at 6 V. Another interesting discovery for the CuI‐based heterojunctions is the piezoelectric enhancement of ZnO (Figure 8d).^[^ ^77^ ^]^ As an alternative approach to chemical doping, the construction of a p–n heterojunction has been demonstrated as an effective strategy to enhance the piezoelectric energy harvesting of ZnO nanomaterials. For a single ZnO piezoelectric nanogenerator, the release of pressure could dramatically decrease the piezoelectric potential with low output performance. The employment of a p‐type CuI layer can largely weaken the electron screening effect. The maximum output voltage and current of 5 V and 1800 nA cm^−2^, respectively, were achieved with a 280 nm thick CuI film. The further increase in the CuI thickness resulted in a performance degradation because of the unfavorable interface.

### 3.4 Thin‐Film Transistors

Owing to the intrinsically poor hole transport property of conventional p‐type metal oxide semiconductors, more attention has been paid to CuI to realize high‐performance p‐channel transistors for CMOS circuit integration.^[^ ^44^, ^72^, ^101^, ^102^, ^105^, ^147^, ^148^, ^149^ ^]^ The first CuI TFT was reported by Choi et al. in 2016 using ink‐jet printing at a processing temperature of 150 °C.^[^ ^105^ ^]^ The optimized TFTs exhibited an average field‐effect mobility (*μ*_FE_) of 1.86 cm^2^ V^−1^ s^−1^ and *I*_on_/*I*_off_ of 10–10^2^. Thereafter, our group conducted a systematic investigation on the spin‐coated CuI TFTs and highlighted that the CuI channel layer thickness, postannealing temperature, and solvent selection were crucial for achieving good device performance.^[^ ^44^ ^]^ The 5 nm thick CuI channel layer deposited at RT enabled an optimized electrical performance with a *μ*_FE_ of 0.4 cm^2^ V^−1^ s^−1^, *I*_on_/*I*_off_ of ≈10^2^, and good reproducibility. The further integration of a complementary inverter with n‐type oxide TFTs exhibited rail‐to‐rail voltage inversion with a voltage gain >4, which demonstrates the great potential of CuI for applications in electronic devices. Gong and co‐workers then employed solid iodized CuI channel layers in TFTs and demonstrated a high linear *μ*_FE_ of 4.8 cm^2^ V^−1^ s^−1^ with an *I*_on_/*I*_off_ of 10–10^2^.^[^ ^72^ ^]^ By employing a high‐capacitance solid polymer electrolyte as a dielectric layer, Lee et al. realized CuI TFTs operating at very low voltages and their combination with n‐channel ZnO TFTs achieved a complementary inverter with sharp voltage transition and a high gain voltage of ≈18.^[^ ^101^, ^102^ ^]^ Meanwhile, the authors utilized vacuum annealing and iodine incorporation approaches to create the *V*_Cu_‐ or *V*_i_‐rich CuI and revealed variations in the electrical properties. In these reports, the anomalously high *μ*_FE_ ranging from 10 to 90 cm^2^ V^−1^ s^−1^ were reported. Considering the moderate Hall mobilities (30–50 cm^2^ V^−1^ s^−1^) of CuI channel layers and the 44 cm^2^ V^−1^ s^−1^ mobility for a single‐crystal CuI, the explanations for these high *μ*_FE_ values are worthy to be clarified in detail. It is noted the high‐capacitance electrolyte dielectric was employed as the gate insulator. In this case the *μ*_FE_ should be carefully evaluated to avoid the underestimation of gate capacitance and thus the overestimation of *μ*_FE_.

**Figure 9:**
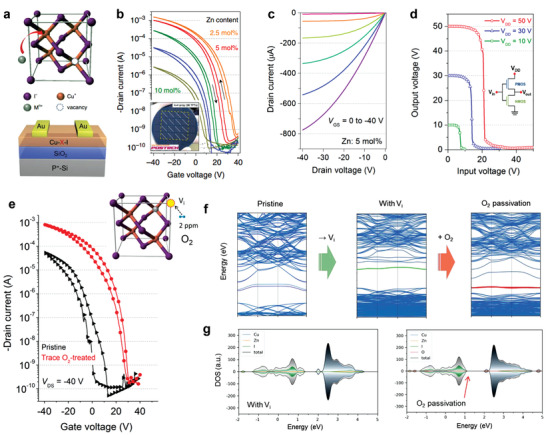
a) Schematic illustrations of a unit‐cell of CuI and the TFT structure. b) Transfer curves of CuI:Zn/SiO_2_ TFTs as a function of the Zn^2+^ content (*V*_DS_ = −40 V). c) Output curves of the optimized TFTs and d) voltage transfer curves of the complementary inverter connected with n‐channel IGZO TFTs. Reproduced with permission.^[^ ^45^ ^]^ Copyright 2020, Nature Publishing Group. e) Transfer curves of Zn‐doped CuI TFTs before and after trace O_2_ treatment. f) Band structure of neat CuZnI, with *V*_i_ and O_2_ occupation on *V*_i_ and g) PDOS of CuZnI with *V*_i_ and after O_2_ occupation. Reproduced with permission.^[^ ^150^ ^]^ Copyright 2021, Wiley‐VCH.

Despite the initial success of CuI as the active layer in p‐channel transistors, the excessive hole density resulted in poor current modulation with a high off‐state current and a low *I*_on_/*I*_off_. To address this issue, our group recently proposed a Zn^2+^ substitutional doping route to achieve high‐performance and reliable CuI TFTs with a high *μ*_FE_ of ≈5 cm^2^ V^−1^ s^−1^ and a record high *I*_on_/*I*_off_ up to 10^7^ at a low process temperature of 80 °C.^[^ ^45^ ^]^ The theoretical calculations indicated that Zn^2+^ was the optimal n‐type dopant for CuI because of its suitable ionic size and similar crystalline texture (**Figure** **9**). Doping with 5 mol% Zn^2+^ improved the film crystallinity, optical transparency, and surface morphology. This was attributed to the passivation effects of ZnI_2_ and retardation of the fast crystallization of CuI. The TFT transfer curves showed a clear negative *V*_TH_ shift with a reduced *μ*_FE_ after Zn^2+^ incorporation, which is a typical n‐doping characteristic (Figure 9b). In addition to the good electrical performance, the CuZn_5mol%_I TFTs delivered band‐like transport properties, excellent reproducibility, and large area uniformity. The large linear current at low drain voltages from the output curves indicated the Ohmic contact between the Au electrodes and the CuZnI channel layer (Figure 9c). Finally, a high‐performance complementary inverter combined with an n‐channel IGZO TFT was realized with a significantly high peak gain of 56 and a low power consumption of 0.25 µW (Figure 9d).

To deposit the CuI channel layer using a solution process, good device performance could only be obtained in dry air (relative humidity ≤ 20%) or an inert atmosphere. Humidity has a significant effect on the film formation and properties in ambient solution processing.^[^ ^149^ ^]^ To achieve CuI‐based TFTs with high reproducibility, film deposition in an inert glove box is preferred to avoid moisture invasion. However, owing to the low formation energy of *V*_i_, *V*_i_ is easily generated during film processing, having a detrimental influence on the TFT performance and stability. In a follow‐up study, we revealed the key roles of trace O_2_ in CuI as a trap passivator and p‐dopant.^[^ ^150^ ^]^ The as‐deposited CuZn_5mol%_I TFTs were stored in a N_2_‐filled glove box with trace amounts of O_2_ (<2 ppm). The device performance gradually increased with a 3× *μ*_FE_ and reduced hysteresis (Figure 9e). Density functional theory calculations showed that the O_2_ molecules tend to occupy *V*_i_ and capture the minority electrons in the channel layer because of their large electronegativity (Figure 9g,h). This study provided us with an important inspiration that *V*_i_ passivation is crucial for achieving high‐performance CuI‐based electronic devices. The *V*_i_ is well‐known as the migration path for iodide ions.^[^ ^151^ ^]^ In the following study, the specific investigation on ion migration of CuI‐based films and the associated device performance is needed. In addition to the I^−^ migration, the high diffusivity of Cu^+^ is observed in Cu(I)‐based semiconductors.

### 3.5 Solar Cells

**Figure 10:**
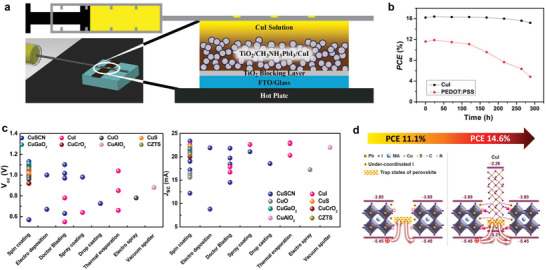
a) The first CuI HTL‐based perovskite solar cell using the drop‐casting method. Reproduced with permission.^[^ ^156^ ^]^ Copyright 2014, American Chemical Society. b) The PCE variations for unencapsulated perovskite solar cells with CuI or PEDOT:PSS HTLs as a function of air exposure time. Reproduced with permission.^[^ ^157^ ^]^ Copyright 2016, Royal Society of Chemistry. c) Statistical results of open‐circuit voltage and photocurrent density for the solar cells with different inorganic Cu‐based HTL layers deposited using different methods. Reproduced with permission.^[^ ^164^ ^]^ Copyright 2019, Elsevier. d) Schematic illustration of the CuI passivation mechanism on the perovskite grain boundary. Reproduced with permission.^[^ ^163^ ^]^ Copyright 2017, American Chemical Society.

Owing to the high hole conductivity of CuI and the well‐aligned VBM with the emerging solar absorber layers, for example, methylammonium lead iodide (MAPbI_3_) perovskite, CuI has been widely utilized as an inexpensive dopant or promising alternative to organic HTLs in organic or perovskite solar cells, leading to an enhanced power conversion efficiency (PCE) and stability.^[^ ^15^, ^152^, ^153^, ^154^, ^155^ ^]^ The first attempt to incorporate CuI HTLs into solar cells was reported by Christians et al. in 2014.^[^ ^156^ ^]^ A 1.5 µm thick CuI was deposited on a porous TiO_2_/MAPbI_3_ layer by using an automated drop‐casting method at low temperatures and a promising PCE of 6.0% with excellent photocurrent stability was achieved (**Figure** **10**a). Later, the doctor blading approach was adopted by Sepalage et al. for the deposition of CuI HTLs to control the morphology and avoid the dissolution of the perovskite layer during CuI coating.^[^ ^39^ ^]^ The use of CuI instead of commonly used organic spiro‐OMeTAD greatly suppressed the cell hysteresis. One year later, Sun et al. adopted RT solution‐processed CuI HTLs replacing conventional poly(3,4‐ethylenedioxythiophene):poly(styrenesulfonate) (PEDOT:PSS) and achieved a significantly enhanced PCE from 13.6% to 16.8% (Figure 10b).^[^ ^157^ ^]^ The CuI HTL was found to effectively reduce the energy loss at the HTL/perovskite interface, improving charge extraction, and inhibiting charge recombination. The unencapsulated CuI‐involved devices showed better air stability than that of PEDOT:PSS cells and a positive aging effect was noticed with a continuously increased PCE up to 20 days because of the improved interfacial contact. During the same period, several research groups verified the suitability of CuI as an HTL instead of its organic counterparts.^[^ ^40^, ^98^, ^99^, ^103^ ^]^ A high PCE of 17.6% with reduced hysteresis and excellent cell stability was reported by Li et al. in 2017.^[^ ^158^ ^]^ To avoid damage to the underlying MAPbI_3_ perovskite layer, a facile spray‐coating process was used for the deposition of the CuI (60 nm) HTL. The device exhibited constant PCE over 50 days of storage under ambient conditions (8% efficiency reduction after 90 days). The obtained PCE is also one of the highest values ever reported for perovskite solar cells with inorganic HTLs. One year later, Zhang and co‐workers first introduced the CuI HTL into Pb‐free inorganic perovskite, that is, Cs_2_Bi_2_I_9_, solar cells.^[^ ^159^ ^]^ The high PCE of 3.2% remained over 57% after 1 month of storage in an ambient atmosphere (relative humidity = 45% at 25 °C). In addition to the solution process, Wang et al. adopted the vapor iodination approach to grow a uniform CuI HTL and the devices delivered a PCE of 14.7% and an open‐circuit voltage of 1.04 V.^[^ ^160^ ^]^ By partially iodinating Cu nanowires with the formation of CuI/Cu hybrid nanostructures, Cao et al. reported high‐performance inverted perovskite solar cells with a maximum PCE of 18.8%.^[^ ^161^ ^]^ The synergistic effect was achieved by the outer CuI layer accelerating the charge extraction and the inner layer facilitated rapid charge transfer toward the cathode.

In addition to its outstanding application as an HTL in solar cell devices (Figure 10c), CuI was used as an effective additive in HTL and perovskite absorber layers. Li et al. directly added CuI into spiro‐OMeTAD to compose the hybrid HTL.^[^ ^162^ ^]^ The incorporated CuI achieved a p‐doping effect with efficient charge transfer, leading to an improved PCE, fill factor, and short‐circuit current density. The addition of CuI to spiro‐OMeTAD suppressed film aggregation and crystallization with reduced pinholes. The compact structure can inhibit the moisture/O_2_ infiltration and lower the perovskite decomposition to some extent. Ye et al. then explored the incorporation of CuI into the perovskite layer (Figure 10d).^[^ ^163^ ^]^ The results showed that the CuI additive could effectively passivate trap states upon interactions with undercoordinated cations and anions at the perovskite grain surface. Meanwhile, the CuI could form bulk heterojunctions with perovskite, further expediting hole transport and reduced charge carrier recombination.

## 4 Conclusions and Perspectives

The past decade has witnessed tremendous development of CuI TC/S in the field of thin‐film microelectronic devices. Compared to oxide and chalcogenide materials, CuI thin films can be easily synthesized through a variety of methods at plastic‐compatible temperatures. Overall, the thermal evaporation and spin‐coating processes enabled a smooth surface morphology at low deposition temperatures, and the physical vapor film growth approaches achieved higher p‐type conductivity owing to the high film quality. The well‐documented challenges for CuI are the instabilities under O_2_ and moisture attacks. Degradation is generally attributed to recrystallization, grain growth, iodine loss, and undesired CuI oxidation.^[^ ^43^ ^]^ However, more efforts are required to clarify the CuI degradation mechanism in air for CuI stabilization and commercialization. The most direct and effective way is passivating or encapsulating the device to physically isolate it from external stimuli in the surrounding environment. From the material point of view, higher stability is expected with the improvement of film quality or component engineering. The diffusion of O_2_/moisture in polycrystalline samples mainly occurs along the grain boundaries and pinholes. The pristine CuI shows a fast tendency for crystallization and easily aggregates with poor topography. The solvent/additive engineering is expected to produce high‐quality, uniform, dense, and pinhole‐free CuI samples.

For electrode applications, smooth surface roughness is required to maintain high optical transparency. Meanwhile, the iodine‐rich deposition condition is preferred for high p‐type conductivity. To further boost the conductivity comparable to that of n‐type oxide counterparts, the substitution of iodine with chalcogen elements, for example, S^2−^, is feasible and worthy of being pursued. For thermoelectric applications, devices are required to work in harsh environments with large temperature differences or high temperatures. Based on our thermogravimetric analysis of CuI powder, the material has no obvious weight variation below 300 °C in air. However, iodine decomposition (common failure of halide materials) occurs at elevated temperatures. This raises the question that the displaced iodine ions are temporarily dissociated in the lattice and later may return to the original position (reversible) or the iodine turns into a vapor and leaves the material (irreversible). A previous report revealed that encapsulation helped reduce iodine decomposition in perovskites and the feasibility should be explored in a CuI‐based system. For transistor applications, despite the initial attempt, CuI has shown great superiority in terms of device performance and processing simplicity as compared to conventional p‐type metal oxides and CuSCN. Future studies should pay more attention to stability issues. One is the long‐term bias‐stress stability under imposed temperature, light illumination, and different environments. In our recent study, we found that *V*_i_ was easily generated during the film deposition and postannealing process. *V*_i_ acts as an electron donor and its passivation is important to achieve high‐performance devices. Another stability issue is the development of a suitable passivation material or encapsulation technique to guarantee robust device operation in air. A multichannel configuration instead of a single channel layer has achieved great success in the performance and stability improvement of n‐type oxide TFTs and is worthy of pursuing for the CuI system.^[^ ^165^, ^166^, ^167^ ^]^ Additionally, considering the air‐sensitive feature of CuI, the development of a feasible patterning process and materials are crucial for circuit integration and more functional applications. Vacuum deposition approaches can use masks to achieve the pattern process while the solution processes require template‐assisted patterning technologies or ink‐jet printing to realize the integrated device array.^[^ ^168^ ^]^ The effect of dielectric environment on the charge transport property of CuI is still unknown. For the inorganic metal oxide and chalcogenide semiconductors, the increment of gate capacitance shows positive effect on the improvement of device *μ*_FE_.^[^ ^169^, ^170^, ^171^, ^172^ ^]^ As the HTL for solar cells, the cheap CuI correlates to low production costs and its intrinsically high mobility and conductivity do not require any additives or dopants, making the optimization process simpler and devices more stable. Preliminary experiments already demonstrated the superior long‐term stability when compared to the cells using organic HTLs and we expect that CuI will play important roles in the achievement of stable, high‐efficiency solar cells in future work.

It is worth noting that the current applications are mainly focused on polycrystalline CuI‐based thin films. For device fabrication with great uniformity over a large area, an amorphous structure is desired and the absence of grain boundaries further enhances the stability. Computational screening and first‐principles predictions are believed to be effective approaches for selecting suitable dopant/alloy elements for the fabrication of amorphous CuI‐based materials with the desired optoelectrical properties (one typical example is an amorphous InGaZnO semiconductor). Ultimately, the knowledge will be shared between computational materials science and experimental synthesis assisted by state‐of‐the‐art characterization techniques that will enable us to connect experiments with predictions and integrate the best material into diverse devices for maximum impact.

## Conflict of Interest

The authors declare no conflict of interest.
